# Hidradenoma papilliferum of the vulva: a dermoscopic challenging diagnosis^⋆^

**DOI:** 10.1016/j.abd.2021.11.014

**Published:** 2023-03-24

**Authors:** Vincenzo De Giorgi, Biancamaria Zuccaro, Flavia Silvestri, Federico Venturi

**Affiliations:** Section of Dermatology, Department of Health Sciences, University of Florence, Florence, Italy

Dear Editor,

A 50-year-old woman presented with an asymptomatic cutaneous papular lesion on the vulva (right interlabial fold) of unknown duration. Genital examination revealed a single, firm, well-circumscribed, smooth surfaced focally ulcerated papule (4 × 4 mm) with no pigment (Fig. 1A). There was no evidence of any bleeding, breakdown, or infection. The inguinal lymph nodes were not enlarged on either side. Dermoscopy showed a central reddish ulceration with undermined edges due to the detachment of the mucosal surface from the lower layers surrounded by a whitish halo in the absence of other dermoscopically-relevant parameters (Fig. 1B). There was a non-specific vascular pattern with tiny linear vessels on a reddish and whitish background. The clinical and dermoscopic features did not suggest any diagnosis. If the dermoscopic aspect could be used to exclude a melanocytic lesion, even an achromic one, then a diagnosis of squamous cell carcinoma ‒ a very frequent tumor in this particular anatomical site ‒ could not be excluded. An excisional biopsy was performed followed by a histopathological examination. Microscopically, a dermal cystic adnexal tumor composed of numerous papillary projections lined by a peripheral layer of myoepithelial cells and a luminal layer of tall columnar cells showing decapitation secretion (Fig. 2) was observed; thus, a final diagnosis of hidradenoma papilliferum (HP) was made.

**Figure 1:**
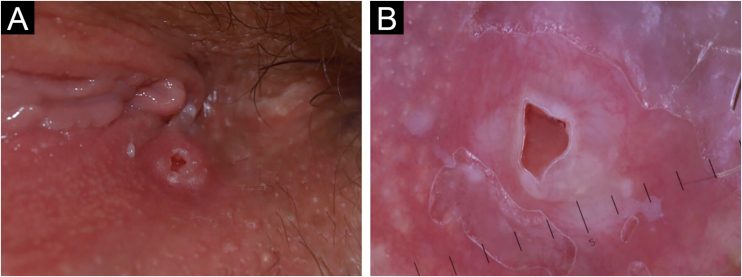
50-year-old woman, right interlabial fold of the vulva. (A) Clinically, single, firm, well-circumscribed, smooth surfaced focally ulcerated nodule (4 × 4 mm) with no pigment. (B) Dermoscopy showed a central reddish ulceration with undermined edges due to the detachment of the mucosal surface from the lower layers surrounded by a whitish halo in the absence of other dermoscopically-relevant parameters.

**Figure 2:**
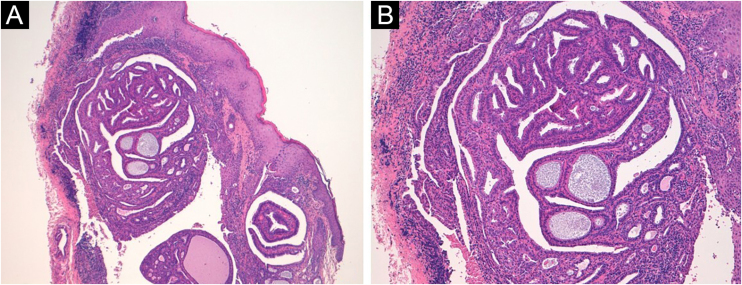
(A) Histopathological examination shows a dermal cystic neoplasm characterized by papillary projections (Hematoxylin & eosin, ×50). (B) Papillae are lined by myoepithelial cells (at the periphery) and tall columnar cells, showing decapitation secretion (Hematoxylin & eosin, ×100).

The patient received no further therapy and had an uneventful clinical course during a 10-year follow-up. During this period, the patient underwent a shave biopsy of another mucosal lesion on the labia minora of the vulva, and the histopathological examination revealed a fibroepithelial polyp with associated mild viral-related changes on the overlying epithelium.

HP is a benign adnexal neoplasm that develops almost exclusively in women with a wide age range preferentially in the labium majus of the vulva. Although HP was previously thought to have an apocrine origin, recent studies have suggested that it may derive from the anogenital mammary-like glands.^1^ It usually presents clinically as an asymptomatic, slow-growing, well-circumscribed, flesh-colored to red nodule typically located in the vulval interlabial sulcus or labium majus.

The term “ectopic” HP has been reported to describe cases occurring in the head and neck as well as the breast, axilla, external ear canal, and eyelid.

HP may have a heterogeneous clinical appearance and can mimic other vulval neoplasms; thus, the ﬁnal diagnosis needs to be conﬁrmed histologically.

Although HP has been histologically well characterized, to the best of our knowledge, the dermoscopic features of vulval HP have only been reported by Tosti et al.^2^ They reported that most polymorphous dermoscopic findings do not lead to a definitive diagnosis. In fact, in the descriptions of the dermoscopic parameters, we found central brown to greyish-blue non-pigmented areas surrounded by dotted and irregular serpiginous telangiectasias or without a recognizable vascular pattern with some shiny white areas. Some lesions were ulcerated. Importantly, the dermoscopic examination can certainly be useful for their differential diagnosis even if we do not have pathognomonic dermoscopic features for the diagnosis of HP and in general for many adnexal neoplasms.^3^

In fact, dermoscopically, the diagnosis of vulvar melanoma is characterized by the presence of more colors, i.e., blue, gray, white, and red color. Furthermore, we often see a whitish veil, especially in thinner vulvar melanomas.^4^ These lesions are also often clinically bleeding and painful.

Vulvar SCC is also common and is characterized dermoscopically by the presence in the initial forms of very compact irregular white areas that do not facilitate any vascular pattern. In our case, the white areas are less defined and intense allowing the vision of even a delicate vascular pattern.

Vulvar basal cell carcinoma is characterized by poor pigmentation but frequently by the presence of blue ovoids that are not present in our case.^5^ Therefore, a careful dermoscopic analysis of the lesion does not offer a precise diagnosis in these cases but allows us to better orient ourselves in the preoperative diagnosis of these lesions in this particular site including those that enter into differential diagnosis with adnexal tumors and therefore with HP.

The overall prognosis for HPs is good. Local excision is the treatment of choice. Tumor recurrence is unusual and is typically attributed to incomplete excision of the primary tumor. Malignant transformation of HP is also rare. In our patients, the neoplasm showed well-defined margins and no recurrence or malignant change during the prospective follow-up period.

In conclusion, HP on the vulvar site is a diagnostically challenging benign entity. The tumor is quite rare, and physicians do not gain sufficient experience to suspect it. Dermoscopy of this lesion, even if not decisive in the diagnosis, can be useful in the differential diagnosis of important neoplasms such as melanoma and SCC. However, in these cases, the diagnostic gold standard remains the histopathology.

## Financial support

None declared.

## Authors' contributions

Vincenzo De Giorgi: Study concept and design; analysis and interpretation of data; drafting of the manuscript; critical revision of the manuscript for important intellectual content; study supervision.

Biancamaria Zuccaro: Acquisition of data.

Flavia Silvestri: Acquisition of data.

Federico Venturi: Acquisition of data.

Vincenzo De Giorgi had full access to all of the data in the study and takes responsibility for the integrity of the data and the accuracy of the data analysis.

## Conflicts of interest

None declared.
